# Radiological and pathological characteristics of giant cell tumor of bone treated with denosumab

**DOI:** 10.1186/1746-1596-9-111

**Published:** 2014-06-07

**Authors:** Michiyuki Hakozaki, Takahiro Tajino, Hitoshi Yamada, Osamu Hasegawa, Kazuhiro Tasaki, Kazuo Watanabe, Shinichi Konno

**Affiliations:** 1 Department of Orthopaedic Surgery, Fukushima Medical University School of Medicine, 1 Hikarigaoka, Fukushima-shi, Fukushima 960-1295, Japan; 2 Department of Radiology, Fukushima Medical University School of Medicine, Fukushima, Japan; 3 Department of Pathology and Diagnostic Pathology, Fukushima Medical University School of Medicine, Fukushima, Japan; 4 Fukushima Pathology Laboratory, Fukushima, Japan

**Keywords:** Giant cell tumor of bone, Denosumab, Neoadjuvant chemotherapy, Receptor activator of nuclear factor-κB ligand (RANKL), Plain radiograph, MRI, ^18^F-FDG PET/CT, Benign fibrous histiocytoma

## Abstract

**Abstract:**

We describe a case of giant cell tumor of the proximal tibia with skip bone metastases of the ipsilateral femur in a 20-year-old man. After the neoadjuvant treatment with denosumab, plain radiographs and computed tomography showed marked osteosclerosis and sclerotic rim formation, and ^18^F-FDG PET/CT showed a decreased standardized uptake value, whereas magnetic resonance imaging showed diffuse enhancement of the tumor, nearly the same findings as those at pretreatment. Pathological findings of the surgical specimen after the denosumab treatment showed benign fibrous histiocytoma-like features with complete disappearance of both mononuclear stromal cells and multinuclear osteoclast-like giant cells.

**Virtual Slides:**

The virtual slide(s) for this article can be found here: http://www.diagnosticpathology.diagnomx.eu/vs/1090602085125068

## Letter to the Editor

Giant cell tumor of bone (GCTB) is a rare, benign primary bone tumor that commonly occurs in young adults. It accounts for approximately 5% of all primary bone tumors and approximately 20% of all benign bone tumors [1-5]. Though categorized as a benign skeletal tumor, GCTB is also known for its locally aggressive behavior and high recurrence rates; 15%–50% after usual curettage only, and 2.3%–20% after curettage with adjuvant treatment (i.e., further debridement with a high-speed burr, cryotherapy with liquid nitrogen, chemical debridement with phenol, or bone cementing) [1,2,4,5]. To improve GCTB’s aggressive course, therefore, new developments in therapy have been sought.

**Figure 1:**
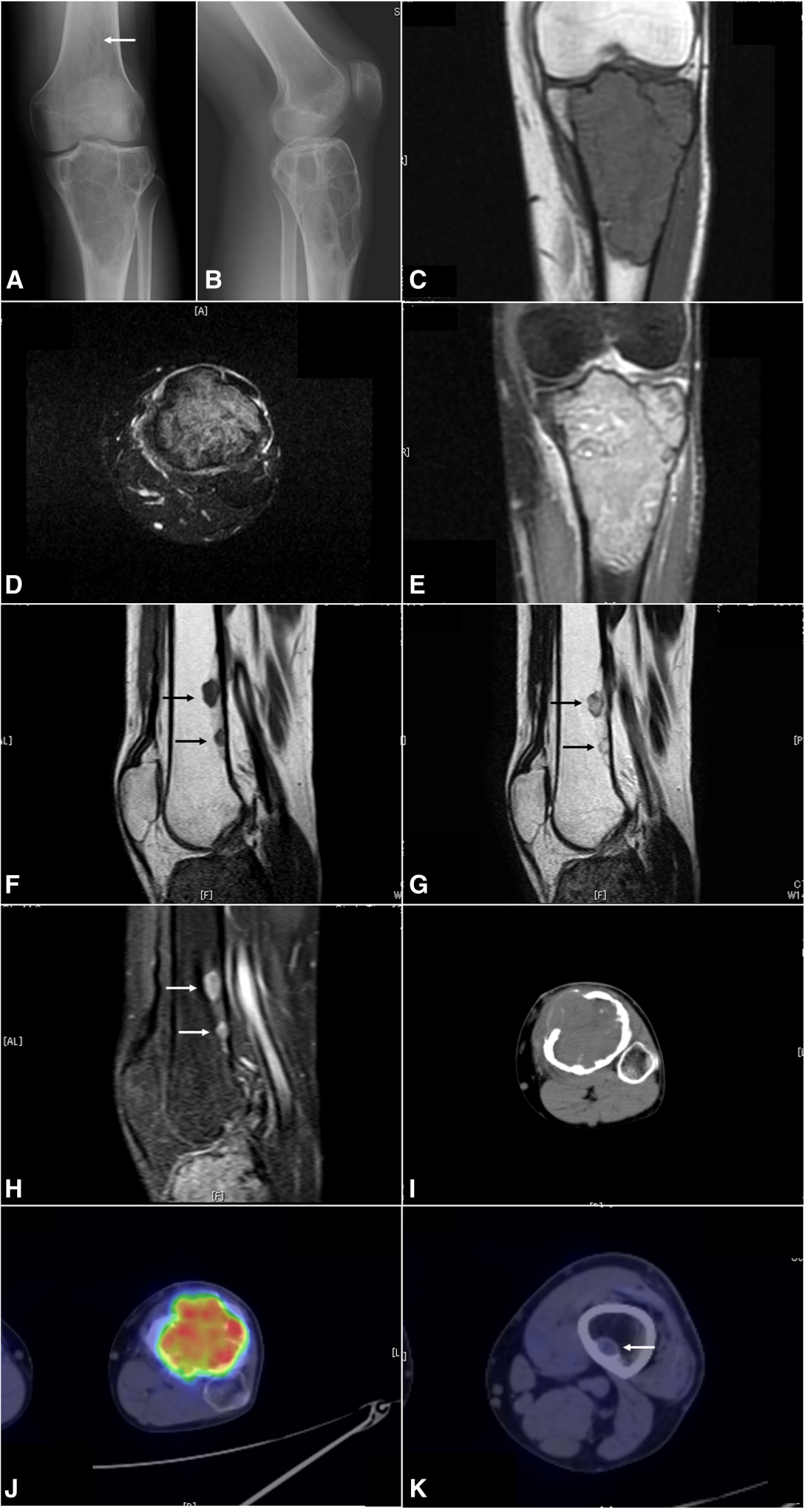
**Pre-treatment radiological analyses of the left knee of the patient.** Plain radiographs show a soap-bubbly osteolytic lesion with thinned cortical bone in the epiphysis of the proximal tibia **(A, B)** and small osteolytic lesions with a nonsclerotic margin in the metaphysis of the distal femur (arrow). MRI shows a proximal tibial tumor displaying iso-intensity to the surrounding muscle on T1-weighted imaging (coronal view) **(C)**, heterogeneous high intensity on T2-weighted fat-suppression imaging (axial view) **(D)**, and diffuse enhancement on gadolinium-enhanced T1-weighted fat-suppression imaging (coronal view) **(E)**. Enhancement of surrounding soft tissue which indicates an occult pathological fracture is also observed. Sagittal MRI of the distal tibia shows small lesions (arrows) displaying nearly the same patterns as the tibial tumor; iso-intensity to the surrounding muscle on T1-weighted imaging **(F)**, high intensity on T2-weighted imaging **(G)**, and diffuse enhancement on gadolinium-enhanced T1-weighted fat-suppression imaging **(H)**. ^18^F-FDG PET/CT revealed the proximal tibial tumor showing marked bone destruction **(I)** with increased SUV uptake (SUVmax: 9.6) **(J)** and the distal femoral lesions with slightly increased SUV uptake (SUVmax: 0.7) (arrow) **(K)**.

Denosumab, the novel monoclonal antibody against receptor activator of nuclear factor-κB (RANK) ligand (RANKL), has recently been used to treat patients with GCTB. Although excellent efficacy of denosumab for cases of advanced or unresectable GCTB has been reported [5-9], the radiological and histopathological findings of GCTB after the denosumab treatment were not described in detail. We describe herein a case of GCTB of the proximal tibia with skip bone metastases, focusing on the radiological and histopathological characteristics observed before and after the preoperative treatment with denosumab.A previously healthy 20-year-old man with a 2-year history of pain in the left proximal lower leg sprained his left knee. After a radiological analysis at the primary hospital, he was referred to our hospital. On admission, the patient noted the pain around his left tibial tubercle both on weight-bearing and at rest. Tenderness and local warmth were observed on the proximal lower leg, and a subcutaneous soft tissue mass was palpable through a defect of cortical bone located just to the outer side of the tibial tubercle. His standard laboratory data showed no abnormalities. Plain radiographs revealed an osteolytic lesion with a soap bubble-like multilocular appearance and thinned cortical bone in the epiphysis of the left proximal tibia (Figure 1A,B). Focal cortical expansion and a partial cortical defect were seen.

Magnetic resonance imaging (MRI) revealed an intraosseous tumor in the left proximal tibia, measuring 9.8 × 6.4 × 5.8 cm in size and displaying iso-intensity to the surrounding muscle on T1-weighted imaging (Figure 1C), heterogeneous high intensity on T2-weighted fat-suppression imaging (Figure 1D), and diffuse enhancement on gadolinium-enhanced T1-weighted fat-suppression imaging (Figure 1E). Positron emission tomography with 2-deoxy-2-[fluorine-18]fluoro- D-glucose integrated with computed tomography (^18^F-FDG PET/CT) showed bone destruction of both cortical and cancellous bone without sclerotic rim (Figure 1I), and an increased standardized uptake value (SUV) on the proximal tibial tumor (SUVmax: 9.6) (Figure 1J).

**Figure 2:**
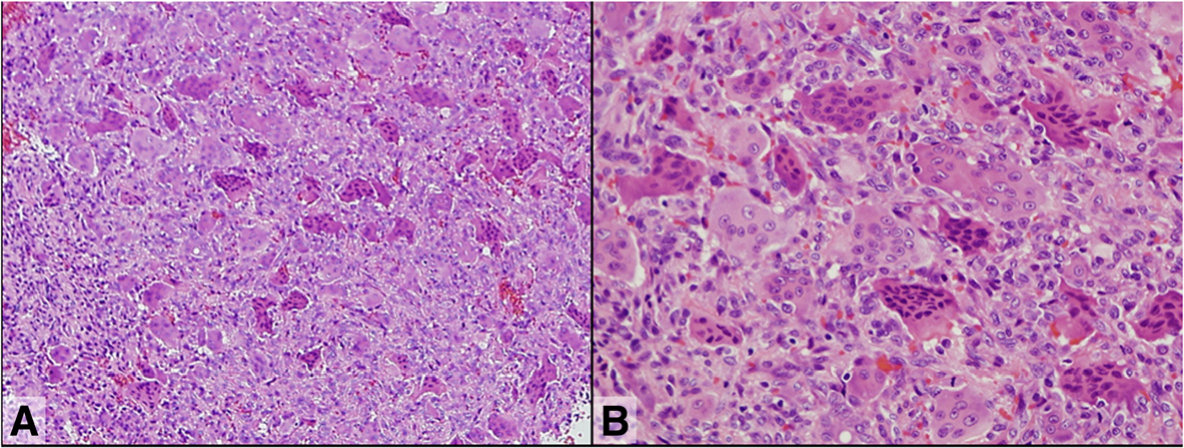
**Histological section of the biopsy specimen (pre-treatment).** The diffuse proliferation of mononuclear stromal cells and osteoclast-like multinuclear giant cells, i.e., typical microscopic findings of GCTB, can be seen (hematoxylin-eosin stain; **A**, ×100; **B**, ×200).

^18^F-FDG PET/CT also detected two small nodular lesions in the distal metaphysis of the left femur (SUVmax: 0.7 and 0.4) (Figure 1K) and no other distant lesion. Plain radiographs (Figure 1A) and MRI of the left distal femur revealed small osteolytic lesions, which showed the same patterns in MRI as the tibial tumor (Figure 1F-H). For the correct diagnosis, we decided to make a histopathological diagnosis via an incisional biopsy of the tibial tumor. The tumor sample was extracted via the cortical defect.Grossly, the tumor was soft, friable, and mixed dark red-brown/yellow tissue which was thought to be compatible with GCTB. Microscopic findings showed a diffuse proliferation of uniform, mononuclear, acidophil cells with oval or short-spindle-shaped nuclei and ill-defined cytoplasm (stromal cells) and osteoclast-like multinuclear giant cells with the similar nuclei as the stromal cells (Figure 2). The mitotic rate was 3/20 high-power field, and atypical mitosis was absent. We diagnosed the tumor as GCTB based on the histopathological findings.

Although we did not diagnose the femoral tumor pathologically, we judged the femoral tumors as skip metastatic tumors from the primary GCTB of the proximal tibia, based mainly on the radiological findings. We decided to treat this patient with denosumab. After dental treatment to prevent osteonecrosis of the jaw, the patient received a hypodermic injection of 120 mg of denosumab at 4-week intervals a total of six times, along with oral calcium lactate (3 g/day) and eldecalcitol (0.75 μg/day). During this treatment, no adverse side effect occurred other than slight hypocalcemia (grade 1, Common Terminology Criteria for Adverse Events [CTCAE] Version 4 [10]).

**Figure 3:**
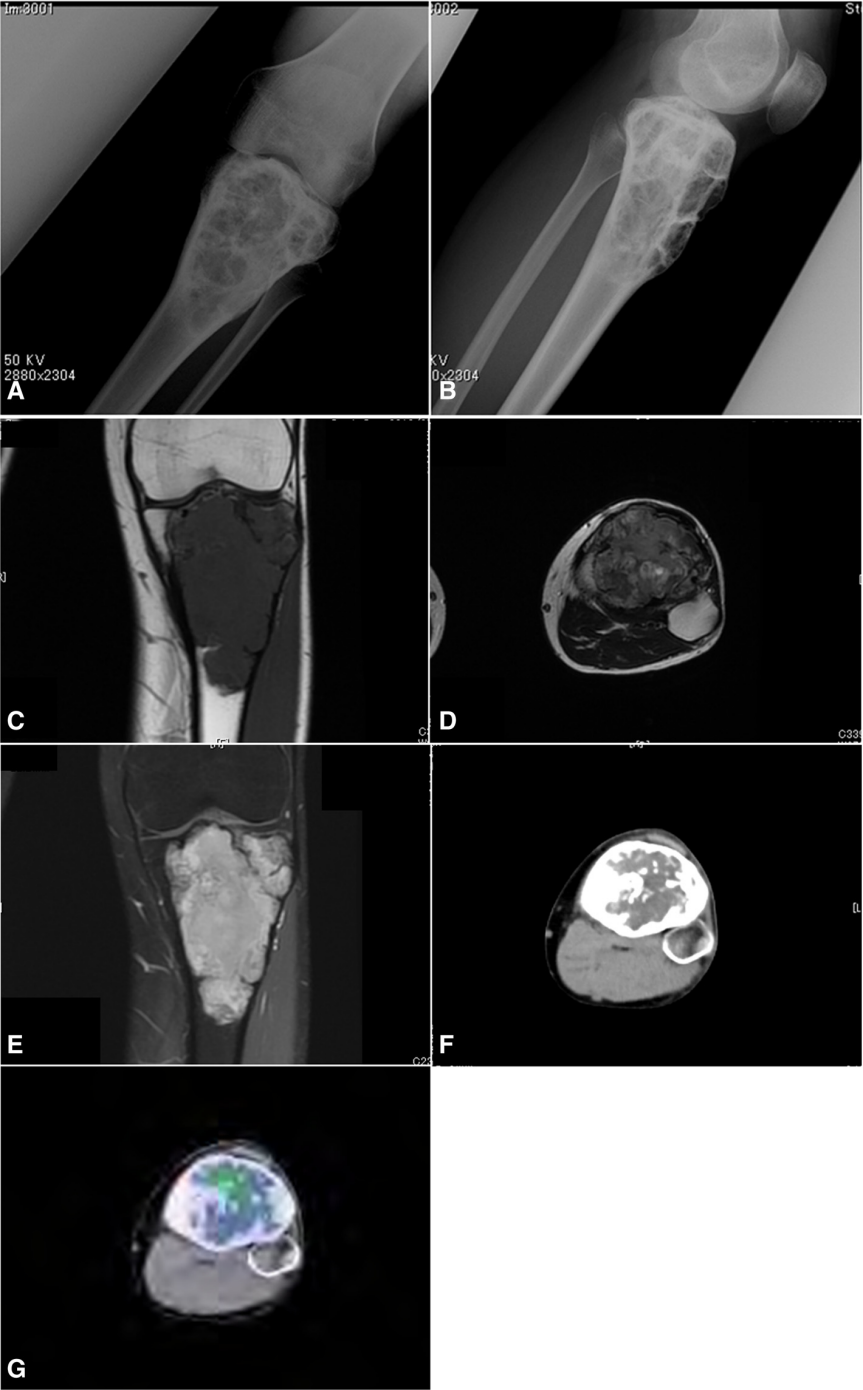
**Post-treatment radiological analyses of the left knee.** Plain radiographs show marked osteosclerosis and sclerotic rim formation on both the proximal tibial tumor **(A, B)** and distal femoral tumors. MRI shows a tumor displaying iso-intensity to the surrounding muscle on T1-weighted imaging (coronal view) **(C)**, heterogeneous high intensity (but lower than at pretreatment) on T2-weighted imaging (axial view) **(D)**, and diffuse enhancement on gadolinium-enhanced T1-weighted fat-suppression imaging (coronal view) **(E)**. ^18^F-FDG PET/CT revealed the marginal/intralesional osteosclerosis of the proximal tibial tumor **(F)** with markedly decreased SUV uptake (SUVmax: 4.8 in the tibial tumor) **(G)**.

The pain around the patient’s left knee disappeared immediately after the first administration of denosumab and he could walk without cane (full-weight bearing). Follow-up plain radiographs were taken every 4 weeks and they showed the progressive osteosclerosis. Six months after the initial denosumab treatment, plain radiographs showed marked osteosclerosis and sclerotic rim formation on both the proximal tibial tumor and the distal femoral tumors (Figure 3A,B). The cortical defect of the left proximal tibia disappeared. ^18^F-FDG PET/CT showed both marginal/intralesional osteosclerosis and osteogenesis (Figure 3F) and a marked decrease in the SUV (SUVmax of the tibial tumor: 4.8, SUVmax of the femoral tumors: 1.3) (Figure 3G). Conversely, MRI showed iso-intensity to the surrounding muscle on T1-weighted imaging (Figure 3C) and diffuse enhancement on gadolinium-enhanced T1-weighted fat-suppression imaging (Figure 3E), which are nearly the same findings as those at pretreatment, and conversely high intensity (but lower than pretreatment) on T2-weighted imaging (Figure 3D). At seven months after the initial medical examination, we performed surgery for the proximal tibial tumor: curettage and further debridement with a high-speed burr, cryotherapy with liquid nitrogen, grafting of beta-tricalcium phosphate, and internal fixation with titanium screws.

**Figure 4:**
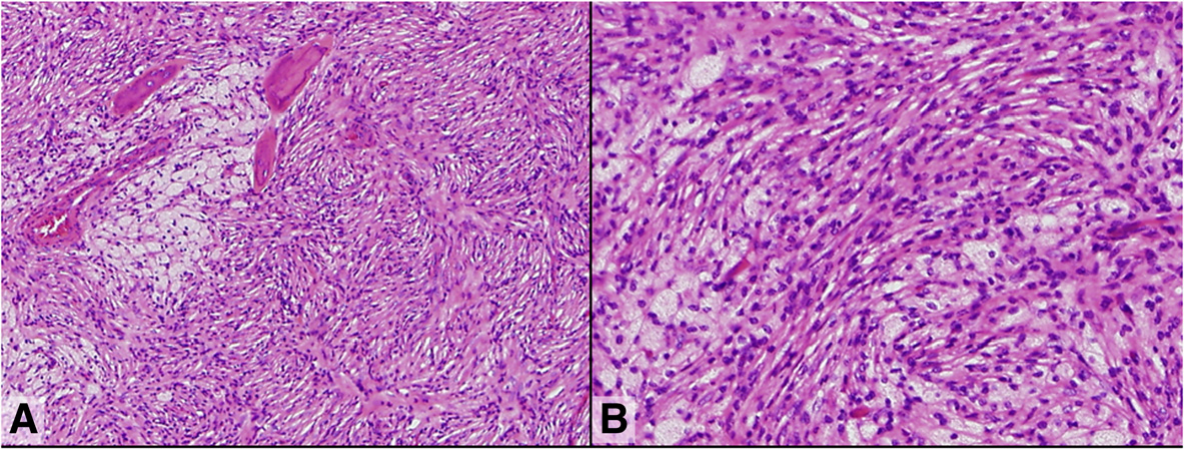
**Post-treatment histological section of the surgical specimen.** The diffuse proliferation of short-spindle-shaped cells arranged in a storiform pattern (mimicking benign fibrous histiocytoma) involving bone formation is shown. Clusters of foam cells are also seen. Both the stromal cells and osteoclast-like giant cells have disappeared. (hematoxylin-eosin stain; **A**, ×100; **B**, ×200).

On gross examination during the operation, we observed thickened cortical bone, peripheral trabecular bone, and internal wall. The tumor was a white, elastic-hard mass which was completely different from the pretreated tumor. Microscopically, the curetted tumor showed diffuse proliferation of short-spindle-shaped cells arranged in a storiform pattern, a benign fibrous histiocytoma (BFH)-like feature (Figure 4), which may be partially observed in some cases of GCTB [11]. While nests of foam cells were scattered, neither stromal cells nor giant cells were observed. The spindle-shaped cells did not have atypical nuclei, and mitotic figures were absent. Partial reactive bone formation was observed. We histopathologically diagnosed the surgical specimen as a post-therapeutic BFH-like lesion after the denosumab treatment for GCTB.

From the post-therapeutic findings of the proximal tibial tumor, there seemed to be BFH-like tissue with no viable stromal cells or giant cells in the femoral tumors. Thus, we decided not to perform surgical treatment on the femoral tumors and to continue radiological observation only.

The patient’s postoperative course was uneventful, and a plain radiograph taken six months after the operation revealed bone union and consolidation without findings of local recurrence. The radiological findings of the femoral tumors showed no remarkable changes after the operation.

GCTB is histologically characterized by the diffuse growth of RANKL-positive mononuclear stromal cells and RANK-positive osteoclast-like giant cells [12]. Since RANKL is a key mediator of osteoclast activation, the RANK-RANKL interaction in GCTB is thought to participate in the growth of the tumor cells, possibly as a result of the production of growth factors by osteoclast-like giant cells through a paracrine loop [8,13]. The inactivation of osteoclasts by denosumab, a human monoclonal antibody that specifically inhibits RANKL, disturbs the bone destruction in patients with osteoporosis [14] and in malignant bone tumors, such as multiple myelomas [15] and metastatic bone tumors [16]. In light of its mechanism of action, clinical efficacy of denosumab for GCTB had been expected.

Since the first report of the efficacy of denosumab for GCTB by Thomas and colleagues in 2010 [6], several studies about this new treatment for GCTB have been published. In these reports, the efficacy of denosumab was evaluated mainly by pathological findings; i.e., the disappearance or decreased number of stromal cells and giant cells, apoptosis or necrosis of tumor cells, fibrosis or increased fibro-osseous tissue, and osteogenesis [7-9,12]. However, these reports did not provide radiological findings, especially in a comparative analysis with radiological and pathological findings.

In the present study, we evaluated the efficacy of denosumab for GCTB both radiologically and pathologically. Our comparative observation demonstrated that the marked osteosclerosis and sclerotic rim formation shown by plain radiographs and CT reflect the devitalization of giant cells and reactive bone formation, and we found that the decreased SUVmax shown by ^18^F-FDG PET/CT relates to the disappearance of tumor cells, mononuclear stromal cells and giant cells.

Conversely, the findings obtained by enhanced MRI pre- and post-treatment were similar, presenting a diffuse proliferation of a BFH-like lesion which was enhanced by gadolinium. Enhanced MRI thus seems to be less useful than plain radiographs or ^18^F-FDG PET/CT for evaluating the efficacy of denosumab treatment for GCTB. However, on plain MRI, T1-weighted imaging was not changed after the denosumab treatment, whereas the intensity of the post-treated tumor on T2-weighted imaging was high (but lower than at pretreatment) in contrast to the circumferential muscle, which was thought to reflect the fibrosis of the tumor. Since the pre- and post-treatment T2-weighted MRIs were not the same, imaging at pre-treatment was with fat-suppression whereas imaging at post-treatment was without fat-suppression, comparative studies with sufficient numbers of GCTB patients treated with denosumab are needed to test this opinion.

Although we did not perform a biopsy for the femoral lesions and did not diagnose them pathologically, the clinical course of the femoral lesions was compatible to that of the GCTB; the radiological reaction to denosumab treatment was the same as that of the tibial tumor. Because multicentric GCTB is an extremely rare entity [17] and the femoral lesions in the present case were much smaller than the proximal tibial tumor, we diagnosed the femoral lesions as skip bone metastases from the primary tibial GCTB. The long-term prognosis of GCTB treated only with denosumab (without surgical treatment) is not yet known, and thus femoral lesions should be carefully monitored.

In conclusion, we have reported a case of GCTB of the tibia with skip bone metastases to the ipsilateral femur, successfully treated with denosumab administration followed by surgical treatment. Based on the results of our comparative radiological and pathological analysis of the pre-/post-treatment tumor, we found that plain radiographs and ^18^F-FDG PET/CT are useful tools for clinical evaluations of the efficacy of denosumab treatment for GCTB. The present study is preliminary, investigating only one patient; this is a major limitation. Further radiological and pathological investigations using larger numbers of GCTB patients treated with denosumab are necessary.

### Consent

Written informed consent was obtained from the patient for publication of this case report and any accompanying images. A copy of the written consent is available for review by the Editor of this journal.

## Competing interests

The authors declare that they have no competing interests.

## Authors’ contributions

MH operated the patient and wrote the paper. OH assessed response by reviewing patient’s radiological studies. KT and KW assessed response by reviewing patient’s pathological studies. TT, HY and SK were involved in a patient care, manuscript preparation and review. All authors read and approved the final manuscript.
